# When does a new sarcoma exist?

**DOI:** 10.1186/s13569-020-00141-9

**Published:** 2020-09-13

**Authors:** Paolo G. Casali, Angelo Paolo Dei Tos, Alessandro Gronchi

**Affiliations:** 1 Fondazione IRCCS Istituto Nazionale Tumori, Milano, Italy; 2 University of Milan, Milano, Italy; 3 University of Padova, Padova, Italy

Pathologists diagnose cancers by looking at how their morphological, histochemical and molecular characteristics cluster in a single case. Almost always this results in a high probability of a known pathologic diagnosis. Occasionally, clustering falls so far from any known paradigm that a new nosological entity is needed. If so, a new diagnostic label is proposed and possibly endorsed by the medical community, thus entering the “morphology” 5-digit entities of the *World Health Organization* (WHO) *International Classification of Diseases – Oncology* (ICD-O) and one of the *WHO Classification of Tumours* series of books (the WHO “blue books”) [1]. Just as widely known examples in the sarcoma field, liposarcoma, leiomyosarcoma and osteosarcoma are well consolidated entities. The explosion of molecular biology, with the increasing use of massive parallel sequencing, is augmenting our chances to find new clusters and thereby create new potential entities. Probably, few cancers are more exposed thereto than sarcomas. In the last years, in fact, several new entities have been added to the *WHO Classification of Tumours—Soft Tissue and Bone Tumours*, and many others are legitimate candidates [2]. Thus, in the era of “precision medicine”, sarcomas may be a privileged area to speculate about which requirements are needed for a new cancer entity to exist. All this is not just an academic issue. In fact, how we conceptually cluster diseases in medicine matters a lot about how we approach them therapeutically.

Why, amongst cancers, are sarcomas so peculiar? First, they are highly heterogeneous. Only hematological neoplasms do present with so many subgroups. The pathologic heterogeneity of sarcomas was shaped by early pathologists on the basis of morphology. Then, immunohistochemistry confirmed and widened morphological partitioning. Eventually, molecular biology is doing the same today with morphological and immunohistochemical entities [3]. Possibly more important, only to some extent does sarcoma partitioning correspond to an obvious histogenesis, unlike hematological neoplasms (in which, say, a subgroup of lymphomas may well correspond to a given stage in normal development of lymphocytes). By and large, in fact, liposarcoma, leiomyosarcoma and osteosarcoma correspond to obvious normal tissues, but this is not the case for several other sarcoma entities. Synovial sarcoma, Ewing sarcoma or solitary fibrous tumor are examples thereof. In the most recent *WHO Classification of Tumours—Soft Tissue and Bone Tumours*, there is a wide array of soft tissue “tumors of uncertain differentiation”, but others can be found amongst the “fibroblastic and myofibroblastic tumours” or the “so-called fibrohistiocytic tumors” [2]. Indeed, many sarcoma entities which do not correspond to any known normal tissue have a natural history that is so peculiar as to single out them clinically. In other words, a synovial sarcoma, a Ewing sarcoma or a solitary fibrous tumor have clinical behaviors which are as specific as their pathologic aspects. Therefore, there would be no reason to challenge their nosological autonomy.

It is worth noting that the lack of a normal counterpart does not necessarily mean the lack of “any” differentiation, atypical though it may be. Just as an example, a desmoplastic small round cell tumor (DSRCT) does not correspond to any known normal tissue, but displays a pathologic differentiation which is highly specific. Probably, it is no coincidence that it is related to an “early” cellular event crucial to the molecular pathogenesis of the tumor: a fusion transcript activating genes underlying, say, histologic desmoplasia. Desmoplasia is a hallmark of DSRCT, pathologically and clinically, and underlies some features of the natural history of these tumors, such as their forming gross abdominal masses. In the end, some sarcoma entities, though not resembling any normal lineage, may present with a specific new differentiation, possibly related to a known strong molecular pathogenesis. A distinct natural history and a distinct clinical behavior follow. Under several respects, synovial sarcoma, Ewing sarcoma or solitary fibrous tumor recapitulate this paradigm.

Today, precision medicine is all about molecularly characterizing any single case. As from its incepts, the ambition has been to lead to “a new taxonomy of human disease based on molecular biology” [4]. In cancer medicine, therapeutically, molecular characterization can allow to predict sensitivity to treatments, especially “molecularly targeted therapies”. Many hopes were raised by the introduction of these agents in cancer treatment (in the sarcoma field, this has been eclatant with gastrointestinal stromal tumors), although secondary resistance has proven a formidable limiting factor, such that the magnitude of clinical benefit has often been limited. On the other hand, medical oncologists are striving to find strong molecular correlates to the activity of the most promising medical therapies in today’s oncology, i.e. those modulating immunity. This said, the idea of precision oncology is that medical therapies may effectively cut across histologies, as molecular markers do. This is widely called “histological agnosticism”. As a result, the first anticancer agents have now received a histologically-agnostic regulatory approval [5, 6]. An example thereof are agents inhibiting the neurotrophic tyrosine receptor kinase (NTRK), the concept being that a NTRK-inhibitor may be effective against different malignancies as long as they harbor a NTRK gene fusion. Then, however, clinical positioning of the drug will be completely different across diseases, being dictated by which is the natural history of the disease, whether at a given stage of disease a medical therapy needs to be used, which other agents are available etc. Thus, an NTRK-inhibitor will be used, say, as a last-line therapy in one malignancy and as a first-line therapy in another, and so forth. In other words, the regulatory approval of a drug may be histologically agnostic, but its clinical use will never be. Indeed, a molecular factor helpful to select medical therapies should never become the key element to define a disease, powerful though its predictive value may be. This is why one could well challenge the “emerging” entity named “NTRK-rearranged spindle cell neoplasm” in the last *WHO Classification of Soft Tissue and Bone Tumours* [2]. While one may suppose that these tumors are sensitive to the new NTRK-inhibitors, the “blue book” depicts their epidemiology, clinical presentation, natural history and prognosis as widely variable and unspecific vis-à-vis the rest of soft tissue sarcomas. In other words, NTRK fusions are their molecular feature, but there is no specific natural history to the best of knowledge today. On top of that, there is no distinct line of differentiation, nor any reason to regard the molecular alteration as clinically relevant, save for the likely activity of a class of drugs. Many other cancers will be sensitive to these drugs outside sarcomas, that is to say agnostically in regard to histology. On the opposite, a well known entity such as infantile fibrosarcoma is pathogenetically related to a gene fusion involving NTRK3, but clearly displays clinical characteristics which are highly specific (in terms of epidemiology, clinical presentation, evolution, prognosis). Its sensitivity to NTRK-inhibitors is just a complement to all that. Thus, while there is no reason to challenge the existence of a disease called infantile fibrosarcoma, one can hardly find reasons, as of today, to regard NTRK-rearranged spindle cell neoplasm as an autonomous nosological entity. This may be true of other new sarcoma entities.

Our working proposal to the sarcoma community is that new diagnostic entities in the sarcoma field should never reflect just a new cluster of morphological, immunohistochemical and/or biomolecular characteristics. Conceptually, when new clusters are appreciated, an attempt should be made to correlate them with a distinct natural history. It is true that in rare cancers as sarcomas, all the more in ultrarare cancers as virtually all new potential sarcoma entities, the low number of patients may make it difficult to generate relevant clinical data in due time. Thus, it may be useful to assume that some specificity in the natural history of disease will be more likely if the new cluster corresponds to either: (1) a normal line of differentiation; (2) a new clear-cut differentiation, possibly resulting from a known strong molecular pathogenesis. In this case, a provisional label could well be accepted, in order to facilitate clinical data collection. But the simple presence of a molecular characteristic serving as a predictor of sensitivity to any anticancer drug should never be enough. This is by no means an obstacle to the fact that it can help tailor medical therapy in any single case and determine the approval of new agents from the regulatory point of view in a histologically-agnostic manner.

We hope that this point of view may be discussed within the sarcoma community. Possibly, any such discussion about the “extreme” area of sarcomas could also be of help to other cancer-based communities, at a time of precision oncology, which, as a by-product, can generate so many new molecular clusters.
